# Study on oligomerization of glutamate decarboxylase from *Lactobacillus brevis* using asymmetrical flow field-flow fractionation (AF4) with light scattering techniques

**DOI:** 10.1007/s00216-017-0735-6

**Published:** 2017-11-22

**Authors:** Jaeyeong Choi, Seungho Lee, Javier A. Linares-Pastén, Lars Nilsson

**Affiliations:** 1 Department of Chemistry, Hannam University, 1646 Yuseong-daero, Yuseong-gu, Daejeon, 34054 Republic of Korea; 2 Division of Biotechnology, Department of Chemistry, Lund University, Naturvetarvägen 16, 22362 Lund, Skåne Sweden; 3 Department of Food Technology, Engineering and Nutrition, Lund University, 22100 Lund, Sweden

**Keywords:** Glutamate decarboxylase (GAD), Oligomerization, Asymmetrical flow field-flow fractionation (AF4), Multi-angle light scattering (MALS), *Lactobacillus brevis* (*L*. *brevis*), Probiotic

## Abstract

In this work, asymmetrical flow field-flow fractionation (AF4) coupled with UV/Vis, multi-angle light scattering (MALS), and differential refractive index (dRI) detectors (AF4-UV-MALS-dRI) was employed for analysis of glutamate decarboxylase (*Lb*GadB) from *Lactobacillus brevis* (*L*. *brevis*). AF4 provided molecular weight (*MW*) (or size)-based separation of dimer, hexamer, and aggregates of *Lb*GadB. The effect of pH on oligomerization of *Lb*GadB was investigated, and then AF4 results were compared to those from molecular modeling. The *MW*s measured by AF4-UV-MALS-dRI for dimeric and hexameric forms of *Lb*GadB were 110 and 350 kDa, respectively, which are in good agreements with those theoretically calculated (110 and 330 kDa). The molecular sizes determined by AF4-UV-MALS-dRI were also in good agreement with those obtained from molecular modeling (6 and 10 nm, respectively, for dimeric and hexameric from AF4-UV-MALS-dRI and 6.4 × 7.6 and 7.6 × 13.1 nm from molecular modeling). The effects of temperature, salt type, and salt concentration on oligomerization of *Lb*GadB were also investigated using dynamic light scattering (DLS). It was found that the hexameric form of *Lb*GadB was most stable at pH 6 and in presence of NaCl or KCl. The results indicate that AF4, in combination of various online detectors mentioned above, provides an effective tool for monitoring of oligomerization of *Lb*GadB under different conditions, such as temperature, pH, type of salts, and salt concentrations.

**Electronic supplementary material:**

The online version of this article (10.1007/s00216-017-0735-6) contains supplementary material, which is available to authorized users.

## Introduction

**Fig. 1:**
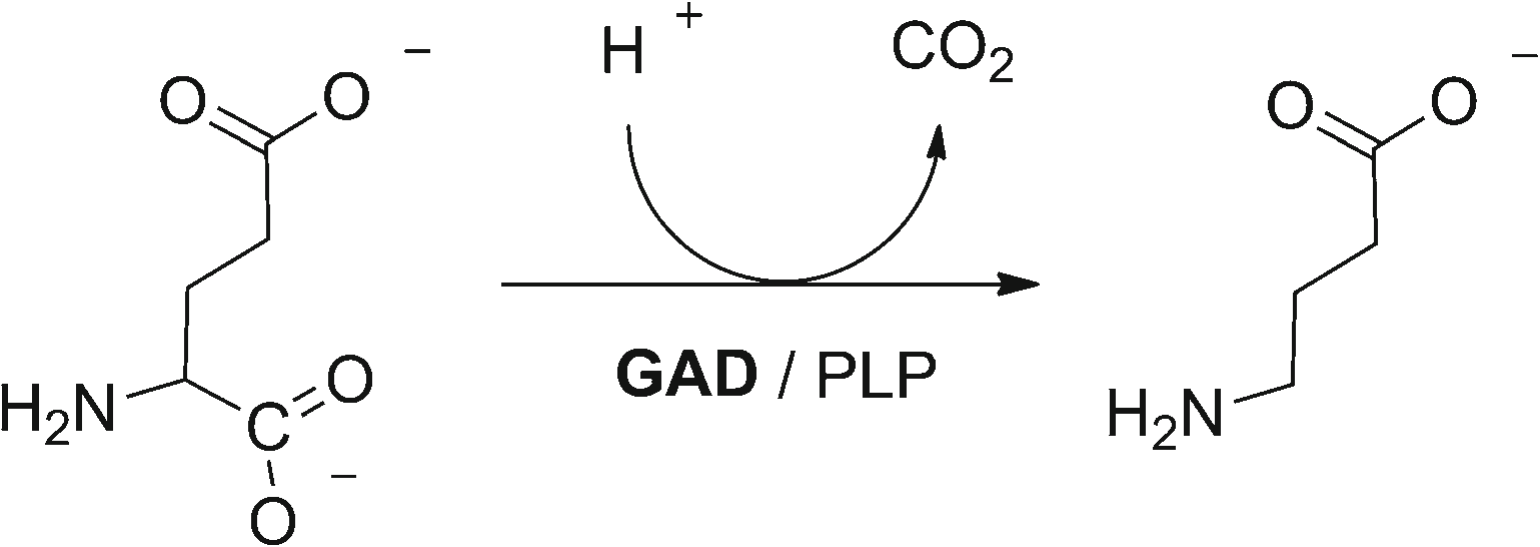
Reaction catalyzed by the glutamate decarboxylase (*Lb*GadB) in the presence of pyridoxal phosphate (PLP) as the co-factor

Glutamate decarboxylase (GAD) catalyzes decarboxylation of glutamic acid giving γ-aminobutyric acid (GABA) (Fig. 1). GAD uses pyridoxal phosphate (PLP) as the co-factor and H^+^ as the co-substrate. This enzyme is present in a variety of organisms, from bacteria to humans. GABA is one of the main neurotransmission inhibitors in the central nervous system. In addition, evidence exist that GABA can lower blood pressure in patients with mild hypertension and that it has other potential beneficial health effects, although mechanisms are not known yet [1, 2]. While GABA is an attractive potential functional ingredient for food, chemically synthesized GABA is not accepted for use in food [3].

GABA is found in some fermented food, as for instance in kimchi [4]. Some strains of *Lactobacillus brevis* (*L. brevis*) were identified as GABA producers [3]. Due to its potential health effects in humans, *L. brevis* is recognized as a putative probiotic [5]. Thus, the potential production of GABA at industrial levels using *L. brevis* as whole cell or its glutamate decarboxylase (*Lb*GadB; notice that “GAD” represents the general name of glutamate decarboxylases present in a variety of organisms, while “*Lb*GadB” represent specifically the glutamate decarboxylase from *L. brevis*) enzyme is very attractive. Therefore, there is great interest to understand the structure and function of *Lb*GadB. It was previously reported that the highest level of activity is reached when GAD is in the hexameric form, while the lowest was observed when GAD was present as a dimer [6, 7]. Thus, oligomerization plays an important role in the GAD mechanism.

In general, size-exclusion chromatography (SEC), gel electrophoresis, and field-flow fractionation (FFF) are used to observe oligomerization of proteins.

SEC is commonly used for measurement of hydrodynamic size (or more commonly molecular weight, *MW*) based on size-based separation of analytes and their size distribution from calibration curve of standard samples or the multi-angle light scattering (MALS) [8, 9]. However, difficulties are frequently encountered when SEC is applied to high *MW* analytes as they may undergo degradation by shear or they may be trapped in the SEC columns [10, 11]. Furthermore, analytes may reach the column exclusion limit or the permeation limit leading to underestimation or overestimation of *MW*, in addition to blockage of the column [11, 12].

Asymmetrical flow field-flow fractionation (AF4) also provides separation of analytes based on their hydrodynamic sizes. In AF4, an open channel without packing material is used [13] and AF4 thus has several advantages over SEC. Due to the relatively gentle separation conditions in AF4 (i.e., low pressure and shear), degradation of analytes is prevented during separation. AF4 has been successfully used for the separation and characterization of high *MW* analytes including proteins, DNA, viruses, and polysaccharides [14–17]. In addition, AF4 provides means to determine some physical properties, without calibration, such as *MW*, size, molecular density, and conformation when it is coupled online with the MALS.

The stability of the active hexameric form of GAD from *L. brevis* depends on several factors, including temperature, pH, salt concentration, and type of salt. Thus, the study of the effect of these factors on the GAD oligomerization is fundamental to optimize the reaction conditions. In this work, the use of diverse techniques, such as AF4 coupled online with MALS (AF4-MALS), dynamic light scattering (DLS), differential scanning fluorimetry (DSF), and molecular modeling methods, allows the integrated characterization of the main physicochemical factors that determine the stability of the oligomeric enzyme GAD.

## Material and methods

### Materials

Citric acid (C_6_H_8_O_7_), sodium hydrogen phosphate anhydrous (Na_2_HPO_4_), sodium chloride (NaCl), potassium chloride (KCl), calcium chloride (CaCl_2_), ammonium sulfate ((NH_4_)_2_SO_4_), and sodium azide (NaN_3_) were purchased from Sigma-Aldrich (St. Louis, USA).The carrier liquid for AF4 was prepared with water purified through a Milli-Q purification system (Millipore Co. Ltd., Billerica, USA, resistance = 18.2 MΩ/cm).

### Molecular cloning

*L*. *brevis* DSM 1269 was purchased from the Leibniz Institute DSMZ-German Collection of Microorganisms and Cell Cultures. The strain was cultured in a MRS (Man, Rogosa, and Sharpe) medium at 30 °C overnight after which genomic DNA was extracted using E.Z.N.A Genomic Isolation Kit (Omega Bio-Tek, USA). PCR primers, specific for GAD gene, were designed based on the genomic sequence of strain ATCC 367: Forward 5′-ATG ACG ACT ATC ATA TGA ATA AAA ACG ATC AGG AAA C-3′ and reverse 5′-GTC AGC TGC CCC TCG AGA CTT CGA ACG GTG GTC-3′ with restriction sites (underlined in the sequences) for NdeI and XhoI, respectively. The amplified gene was inserted in the protein expression vector pET21b giving the construct pET21b::*Lb*GadB, which was introduced in *Escherichia coli* (*E. coli*) Origami 2 (DE3) (Novagen brand, Merck KGaA, Darmstadt, Germany). Recombinant *E. coli* was grown in 500 mL of LB (Luria Bertani) medium supplemented with 100 μg/mL ampicillin, at 37 °C. Recombinant *Lb*GadB production was induced when the culture optical density (OD) at *λ* = 600 nm reaches 0.6, with isopropyl *β*-D-1-thiogalactopyranoside, at 30 °C during 6 h. Finally, the cell pelleted was harvested by centrifugation at 8000×*g* per 10 min in a Sorvall refrigerated centrifuge (RC5C, USA) for the recombinant protein purification.

### Protein purification

Recombinant *Lb*GadB was purified by ion-metal affinity chromatography. *E. coli* cell pellet was washed twice with binding buffer pH 7.4 (50 mM sodium phosphate, 0.5 M NaCl, and 20 mM imidazole). Cell lysis was performed suspending 1 g of cell pellet in 5 mL BugBuster® Protein Extraction Reagent (San Diego, CA, USA) with 5 μL Lysonase™ Bioprocessing Reagent and incubated for 30 min at 25 °C. Next, the suspension was centrifuged at 14,000×*g*, the precipitate was discarded, and the supernatant was injected in a 5-mL HisTrap column FF (GE Healthcare, Uppsala, Sweden) previously equilibrated with binding buffer. After injection, the column was washed with binding buffer and the recombinant protein eluted with elution buffer pH 7.4 (50 mM sodium phosphate, 0.5 M NaCl, and 0.5 M imidazole). Finally, the excess of NaCl was removed by dialysis in 50 mM sodium phosphate buffer at pH 7.4. Protein purity was determined by SDS-PAGE and the concentration was quantified spectrophotometrically at 280 nm.

### Molecular modeling

Hybrid homology model of *Lb*GadB was constructed using the YASARA software [18]. Crystallographic structures of other glutamate decarboxylases deposited in the Protein Data Bank (PDB codes: 3HBX, 1XEY, 1PMM, and 3MAD, with amino acid sequence identities of 38, 39, 38, and 23%, respectively) were used as templates. The modeled structure was refined by molecular dynamic simulation using the AMBER03 force field [19]. The solvent was simulated with explicit molecules of water. Analysis of the modeled structure was done using UCSF Chimera v1.11.2 [20].

### Asymmetrical field-flow fractionation

The asymmetrical flow field-flow fractionation (AF4) used in this work was an Eclipse 3+ system (Wyatt Technology, Dernbach, Germany) coupled online with a UV detector (UV-975, Jasco Corporation, Japan) set at 280 nm, a multi-angle light scattering (MALS) detector (DAWN HELEOS ΙΙ, Wyatt Technology), and a differential refractive index (dRI) detector (Optilab T-rEX, Wyatt Technology). The AF4 channel was trapezoidal with the tip-to-tip length of 26.5 cm and the width at the inlet and outlet of 2.2 and 0.6 cm, respectively, and was equipped with a 350-μm-thick Mylar spacer and a regenerated cellulose (RC) membrane (molecular weight cut-off of 10 kDa, Millipore, Bedford, USA).The AF4 carrier liquid with various pH (3 to 8) was 10 mM citrate-phosphate buffer and was pumped into the AF4 channel using an Agilent 1200 HPLC pump equipped with an auto-sampler and an in-line vacuum degasser (Agilent Technologies, Waldbronn, Germany). The channel flow rate was kept constant at 0.5 mL/min, while the cross flow rate was kept constant at 4.5 mL/min for the first 20 min; after which, it was exponentially decreased from 4.5 to 0.1 mL/min with the half-life time of 2 min and then kept constant at 0.1 mL/min for 30 min. The channel was washed with the carrier liquid for 10 min without cross flow at the end of each run. All AF4 experiments were performed at room temperature. The collection and processing of AF4 data were performed using the ASTRA software (Version 6.1.1, Wyatt Technology) with the *d*_n_/*d*_c_ value of 0.185 mL/g. In all cases, the Berry method was used to fit the light scattering data [21, 22]. From AF4 retention time, the hydrodynamic diameter (*d*_H_) of a sample was calculated by the AF4 theory using the FFFHydRad 2.0 software [23, 24].

### Determination of thermal stability of *Lb*GadB

Thermal stability of *Lb*GadB in different pH values was determined using the Prometheus NT 48 nanoDSF (NanoTemper Technologies, GmbH, Munich Germany). Protein samples were prepared in pH 4, 5, 6, 7, 8 (McIlvaine buffer system, 100 mM), and 9.6 (glycin-NaOH buffer, 100 mM). The capillaries were directly filled with 10 μL of every sample. Intrinsic fluorescence at emission wavelengths of 330 and 350 nm was monitored in a temperature gradient from 20 to 90 °C. All data analysis was performed using the PR control software (Version 2.0, Munich Germany).

### Dynamic light scattering

The dynamic light scattering (DLS) analysis was performed using DynaPro Plate Reader ΙΙ (Wyatt Technology) equipped with a laser with the wavelength of 830 nm as the light source. The analysis conditions were as follows: temperature = 25, 37, 45, and 60 °C; accumulation time = 100 s; and number accumulation = 10. The *Lb*GadB was prepared using various salt types (NaCl, KCl, CaCl_2_, and (NH_4_)_2_SO_4_) at concentrations (0, 0.3, 0.6, 0.9, and 1.2 M). Each sample of 60 μL was put in the supported 384-well plate before DLS analysis.

## Results and discussion

### Protein production

**Fig. 2:**
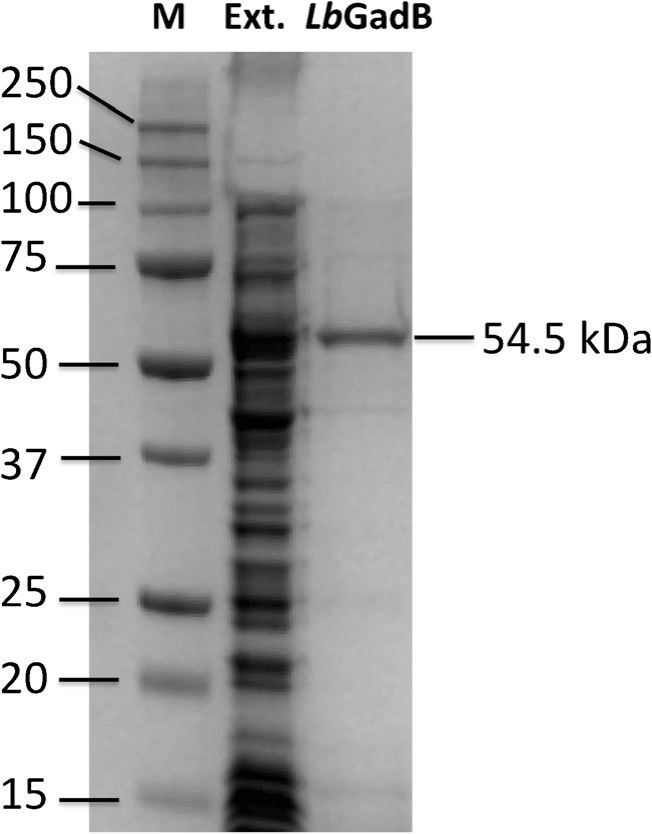
Expression and purification of *Lb*GadB from *L. brevis* DSM 1269

Glutamate decarboxylase gene from *L. brevis* DSM 1269 was cloned and the recombinant protein was successfully produced in *E. coli*. The gene was sequenced and deposited in the GenBank (accession code: KX417371). The protein sequence was obtained by theoretical translation of the gene. The protein sequence was identical to the *Lb*GadB of the strain ATCC 367. The *MW* of the monomer was determined experimentally by SDS-PAGE (see Fig. 2) and theoretically from the protein sequence, giving the consistent value of 54.5 kDa.

### Molecular modeling

**Fig. 3:**
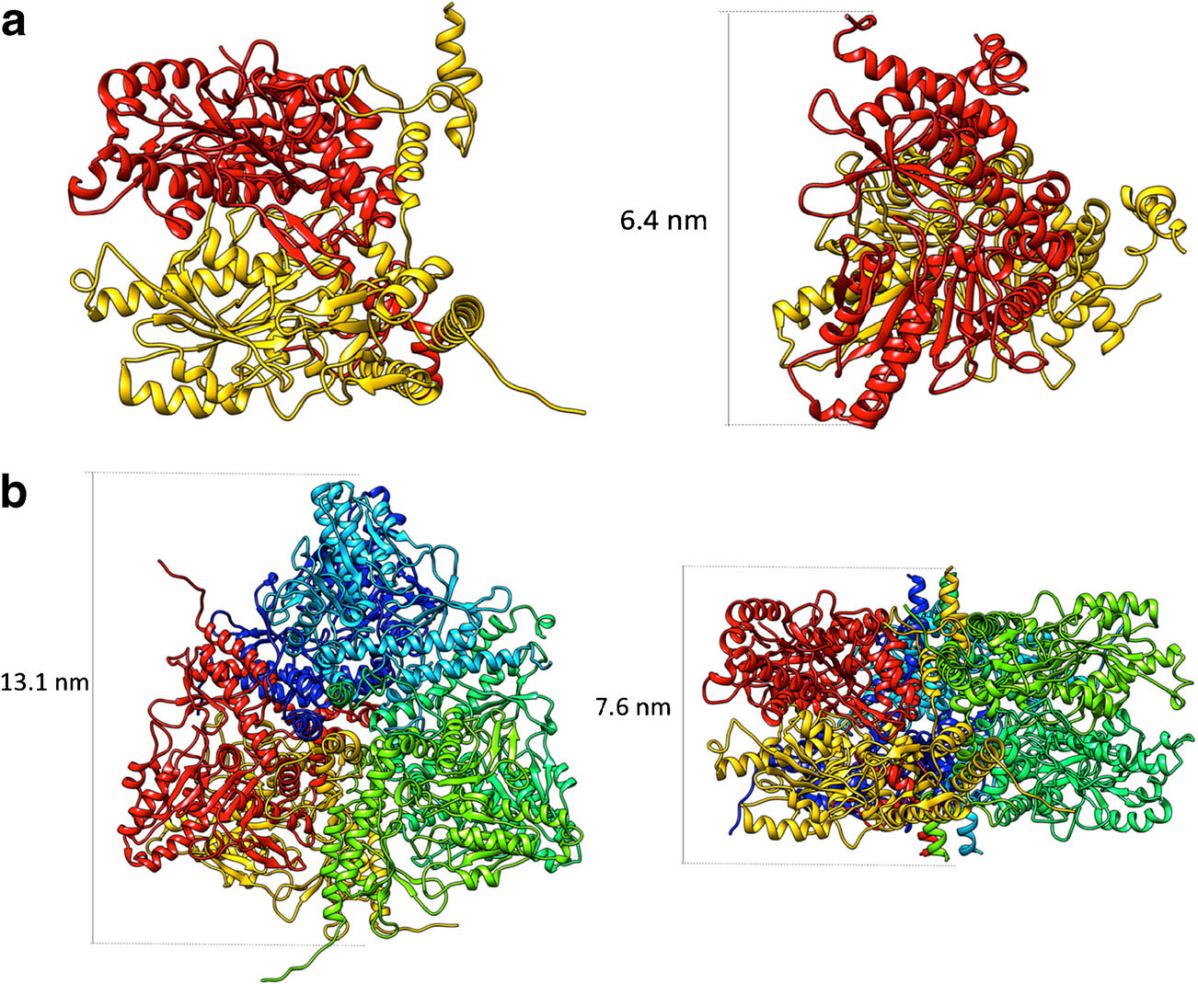
Molecular models of *Lb*GadB: **a** dimer and **b** hexamer

The molecular model was obtained in the hexameric form, which is a trimer of dimers. The overall structure has two layers, where every dimer contributes with one subunit to each layer. Ramachandran plot analysis showed that 94.5% of the amino acids were in the preferred regions, 5% in the allowed regions, and 0.5% were outliers; this suggests that the model is acceptable. The dimer dimension was 6.4 × 7.6 nm, while the hexamer diameter was 13.1 nm and the width was 7.6 nm (Fig. 3).

### Effect of the pH in the oligomerization of *Lb*GadB: AF4 studies

**Fig. 4:**
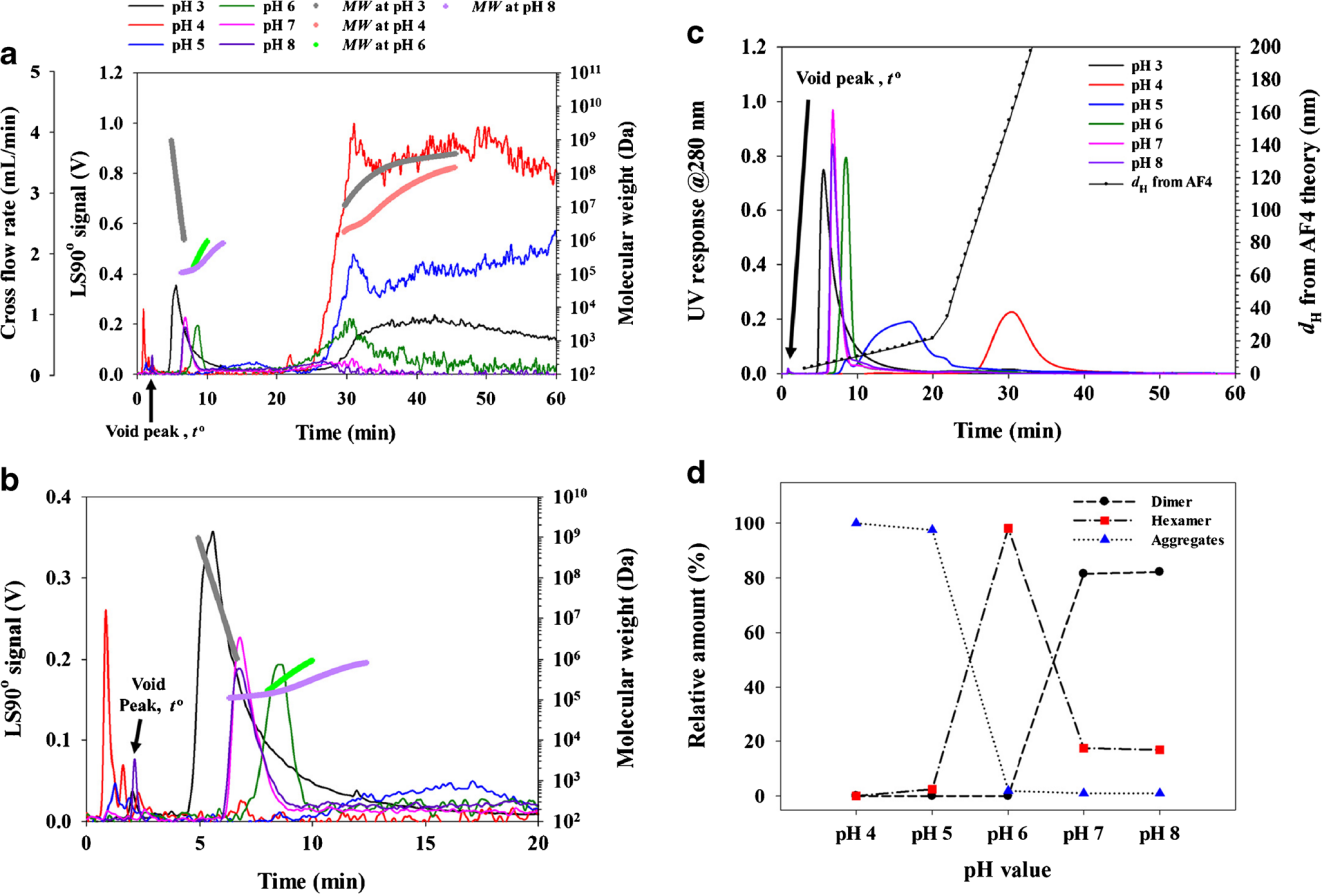
AF4-MALS-UV fractograms of *Lb*GadB obtained at various pH. The injection volume was 200 μL at pH 3 and 4 and 150 μL at pH 5, 6, 7, and 8. **a** LS fractogram at 90° and *MW*. **b** Enlargement of fractogram shown in **a**. **c** UV fractogram at 280 nm and *d*_H_ from AF4 theory. **d** Relative amount of dimer, hexamer, and aggregates of *Lb*GadB measured from peak area of deconvoluted UV fractograms

Figure 4 shows AF4-UV-MALS fractograms of the recombinant *Lb*GadB obtained at various pH. Figure 4a shows the LS responses (measured at 90°) and the molecular weight distributions (*MWD*). Figure 4b shows the same fractograms as those in Fig. 4a at the retention time of 0~20 min. The UV responses are shown in Fig. 4c. As shown in Fig. 4a–c, at pH 7 and 8, the dimers of *Lb*GadB are eluted at ~ 7 min. At pH 6, hexamers are formed, and thus the elution time was increased (~ 9 min). Also a broad band was observed at pH 6 at around 30 min, probably due to elution of *Lb*GadB aggregates. When pH was further lowered down to 5 and then 4, the intensity of the broad band at around 30 min increases, due to more aggregation, with no distinct hexamer peak observed. It is noted, at pH 3, the intensity of the broad band was decreased, and a tailed band was observed at the elution time of around 5 min, which is due to large aggregates eluting in the steric/hyperlayer mode [25, 26]. As shown in Fig. 4b, the molecular weight decreases with increasing time for the tailed band, which confirms the aggregates are eluted by the steric/hyperlayer mode. The average *MW* of the tailed band at 5 min was 5.7 × 10^8^ Da by determined by MALS, which is much higher than the average *MW* (1.1 × 10^5^ Da) of the 7-min peak observed at pH 8.

The *MW* of the dimer and the hexamer of recombinant *Lb*GadB were determined to be 110 and 350 kDa at 10 mM citrate-phosphate buffer of pH 8 and 6, respectively, by AF4-MALS. These *MW* values are in agreement with those measured for the *Lb*GadB monomer, 55 kDa by SDS-PAGE and molecular modeling.

The radius of gyration (*r*_g_) of the dimer and the hexamer of the *Lb*GadB could not be measured by MALS as the analytes behaved as isotropic scatterers, and hence, no angular dependence in the scattered light is observed. The *d*_H_ was determined using AF4 theory [23, 24] and were compared with results obtained from molecular modeling for the dimer and the hexamer of *Lb*GadB. From the AF4 theory, the *d*_H_ of the dimer and the hexamer of *Lb*GadB were determined to be 6 and 10 nm, respectively, and were in reasonable agreement with those from molecular modeling, which were 6.4 × 7.6 nm for the dimer and 7.6 × 13.1 nm for the hexamer. The sizes determined by AF4 theory and modeling are not expected to be identical, as they have different physical meanings, i.e., the *d*_H_ determined by AF4 is the molecular size obtained from the diffusion coefficient (through the Stokes-Einstein equation) [27]. While, the sizes calculated from homology model are based on its atomic three-dimensional structure built using as templates crystallographic structures of homologous proteins [19].

Table 1 shows the *MW* and size determined by various methods for recombinant *Lb*GadB. In Table 1, the *MW* and *r*_g_ were determined by MALS, *d*_H_ were determined by AF4 theory, and molecular dimensions by modeling, respectively.

**Table 1:** Molecular weight (*MW*) and size of recombinant *Lb*GadB determined from peak maximum point of pH 4, 6, and 8 by various methods

Form of *Lb*GadB	Molecular weight (Da)	Radius of gyration, *r*_g_ (nm)	Hydrodynamic diameter, *d*_H_ (nm)	Molecular dimension^a^ (nm)
Dimer	1.1 × 10^5^	n.d	6	6.4 × 7.6
Hexamer	3.5 × 10^5^	n.d	10	7.6 × 13.1
Aggregates	1.8 × 10^6^~2.2 × 10^8^	70~78	80~292	n.d

n.d = no data

^a^Molecular modeling result

In general, proteins show UV absorption at 280 nm due to UV absorbing amino acid, such as tryptophan, tyrosine, and phenylalanine. However, the large aggregates cause scattering effects, so correction of the results is necessary such as deconvolution for quantitative analysis. Thus, all results of UV detector were deconvoluted to dimer, hexamer, and aggregates for semi-quantitative analysis of oligomerization of *Lb*GadB. Figure 4d and Table 2 show the relative percent concentration of the dimer, hexamer, and aggregates of recombinant *Lb*GadB obtained by deconvoluting the UV fractograms in Fig. 4c using the PeakFit software (ver. 4.0, Systat Software Inc., San Jose, USA) with the Savitsky-Golay smoothing.

**Table 2:** Relative concentration of monomer, hexamer, and aggregate of *Lb*GadB obtained by deconvolution of UV fractograms at various pH

pH	Concentration (%)		
	Dimer	Hexamer	Aggregates
4	–	–	100
5	–	3	97
6	–	98	2
7	81	18	1.0
8	82	17	1.0

Consistent with previous studies of plant (*Arabidopsis thaliana*) glucatamate decarboxylase (*At*GAD1) [6], it seems that *Lb*GadB from *L. brevis* is stable in a dimer form at pH 7 or higher. In Fig. 4d and Table 2, only 18% of *Lb*GadB are present in a hexamer form at pH 7. The *Lb*GadB is present mostly as hexamer at pH 6. Then at pH 5 or lower, most of *Lb*GadB are present as aggregates (98% at pH 5 and 100% at pH 4), indicating the hexamer of *Lb*GadB is not stable at pH below about 5. The isoelectric point (pI) of *Lb*GadB calculated by the molecular modeling was 5.15 in the present study and aggregation of *Lb*GadB will be promoted at pH below pI. The hexamer concentration changes drastically from 18% at pH 7 to 98% at pH 6.

**Fig. 5:**
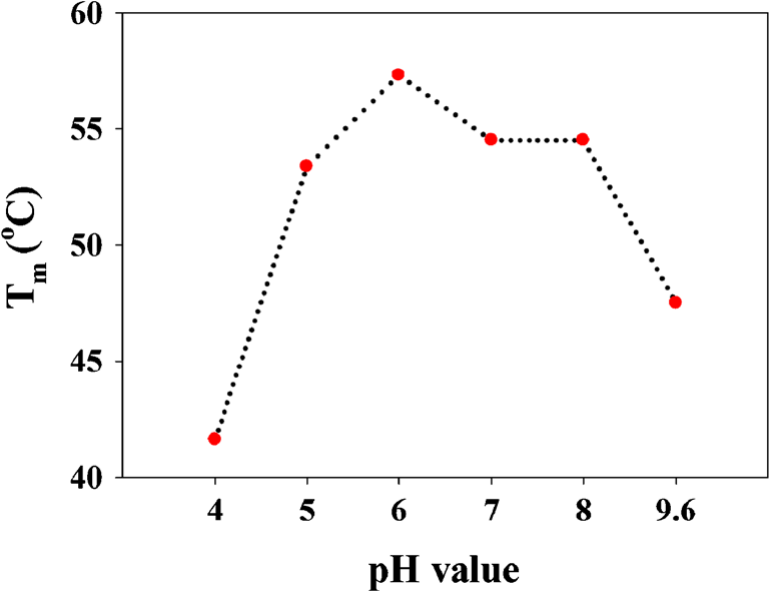
Thermal stability of *Lb*GadB described by the thermal unfolding transition midpoint (*T*_m_) in different pH values

It has been reported that *At*GAD1 is present mostly as hexamer at pH below 7 [6]; in this line, our results show that the hexameric form of *Lb*GadB is more stable at pH 6 than pH 7. Both enzymes, *At*GAD1 and *Lb*GadB, share 38% of amino acid sequence identity (query cover of 94%, BLAST search). The thermal stability of *Lb*GadB in different pHs shows the maximum stability at pH 6 (*T*_m_ 57 °C) and the lowest at pH 4 (*T*_m_ 46 °C) (Fig. 5). At pH 6, the main form is the hexamer, which means that this oligomeric form is the most stable form, while at pH 4 almost all of the protein aggregate (Fig. 4d). At pHs 7 and 8, the predominant form is the dimer, which is less stable than the hexamer since the thermal unfolding transition midpoint (*T*_m_ 54 °C) is lower in both pHs (Fig. 5). The dimer and hexameric forms are stabilized by hydrogen-bonding interactions; therefore, the thermal stability is increased [28, 29]. While, in the case of the aggregates, it is expected that is not thermally stable because it is mostly formed physically with low or without hydrogen-bonding interactions. Thus, the results of AF4 and thermal stability mean *Lb*GadB has most stable form occurs at pH 6. It is new discovery for oligomerization of *Lb*GadB.

### Effect of type and concentration of salt and temperature on oligomerization of *Lb*GadB

**Fig. 6:**
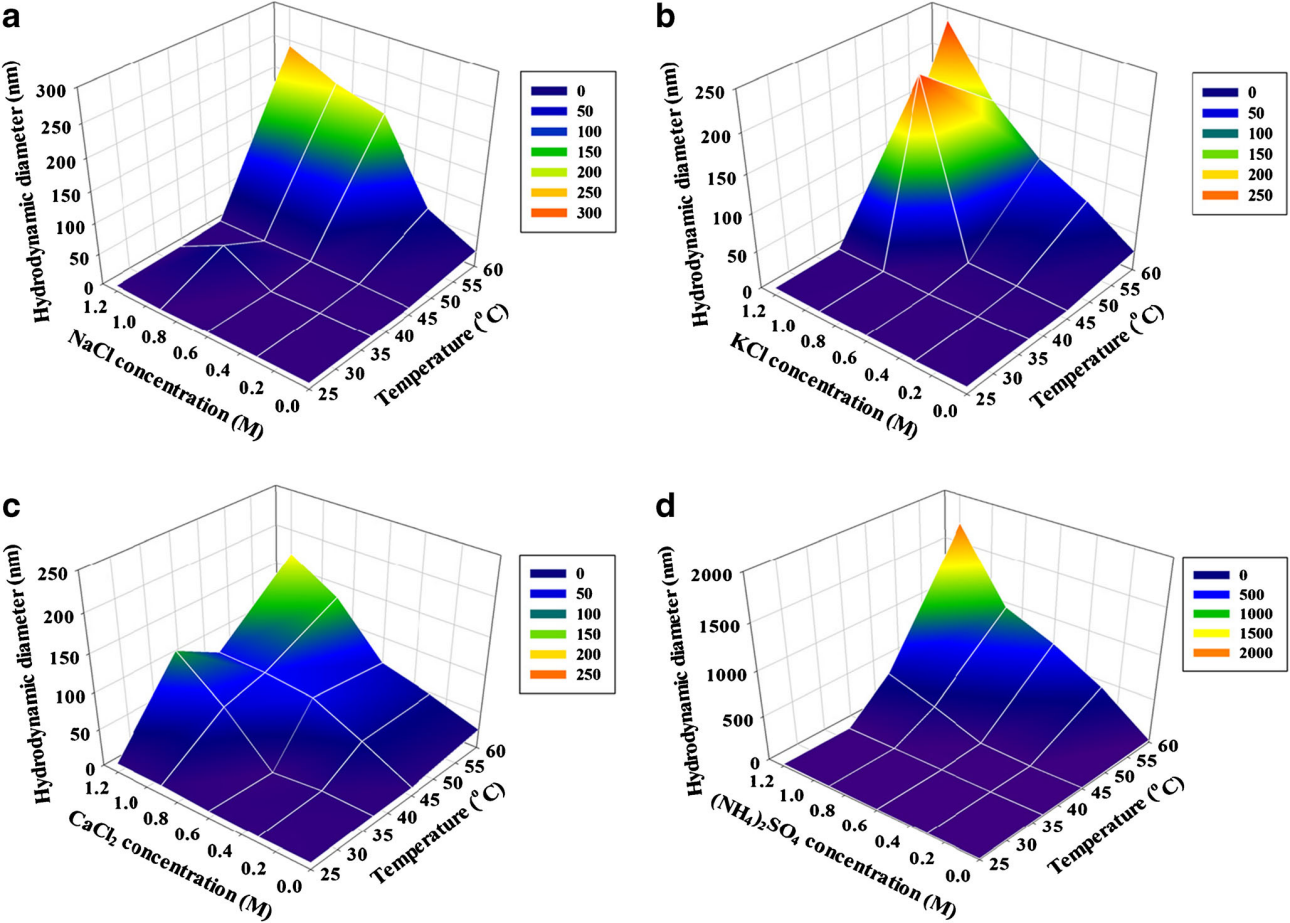
Effect of temperature, salt type, and concentration on oligomerization of *Lb*GadB determined by DLS. **a** Sodium chloride (NaCl). **b** Potassium chloride (KCl). **c** Calcium chloride (CaCl_2_). **d** Ammonium sulfate ((NH_4_)_2_SO_4_) at different temperatures and salt concentrations at pH 7

Figure 6 shows the *d*_H_ of *Lb*GadB determined by DLS with four different types of salts (NaCl, KCl, CaCl_2_, and (NH4)_2_SO_4_) added at five different concentrations (0, 0.3, 0.6, 0.9, and 1.2 M) at four different temperatures (25, 37, 45, and 60 °C). These salts are generally recognized as safe (GRAS) according to Food and Drug Administration (FDA). All results show a similar trend of an increase in *d*_H_ with an increase of salt concentration or temperature. At the lower temperatures (25 and 37 °C), however, no significant changes in *d*_H_ were observed by a change in the salt concentration. Similarly, at the lower salt concentrations (0 and 0.3 M), no significant changes in *d*_H_ were observed by a change in the temperature. The effect by the salt type was not clear. Based on the results at 37 °C (see Electronic Supplementary Material (ESM) Fig. S1 for more details), the hexameric form of *Lb*GadB is most stable in NaCl or KCl and is not affected by the salt concentration in the investigated range.

## Conclusion

The *MW*s and sizes of dimer and hexamer of *Lb*GadB determined by AF4-UV-MALS-dRI were in good agreements with those from molecular modeling. The effects of temperature, salt type, and salt concentration on oligomerization of *Lb*GadB were investigated using DLS. The hexamer content of *Lb*GadB determined by deconvolution of UV/Vis detector response was 98%, indicating the hexamer form is most stable at pH 6. The hexamer also showed high thermal stability (up to about 57 °C). This seems to be a new finding on oligomerization of *Lb*GadB. Results prove that AF4-UV-MALS-dRI is a powerful tool for separation of dimer, hexamer, and aggregates of *Lb*GadB and also for monitoring of oligomerization of *Lb*GadB.

## Electronic supplementary material

ESM 1 (PDF 675 kb)
