# Supplementary material for: Study on oligomerization of glutamate decarboxylase from Lactobacillus brevis using asymmetrical flow field-flow fractionation (AF4) with light scattering techniques
Source: Anal Bioanal Chem. 2017 Nov 22;410(2):451–8. doi: 10.1007/s00216-017-0735-6 (PMC5750328; doi:10.1007/s00216-017-0735-6)
Supplement: Supplementary file 1 — (PDF 675 kb) [file 216_2017_735_MOESM1_ESM.pdf]

Analytical and Bioanalytical Chemistry

Electronic Supplementary Material

Study on oligomerization of glutamate decarboxylase from *Lactobacillus brevis* using asymmetrical flow field-flow fractionation (AF4) with light scattering techniques

Jaeyeong Choi, Seungho Lee, Javier A. Linares-Pastén, Lars Nilsson

Fig. S1 Variation of d_H with salt concentrations at various temperatures and salt types

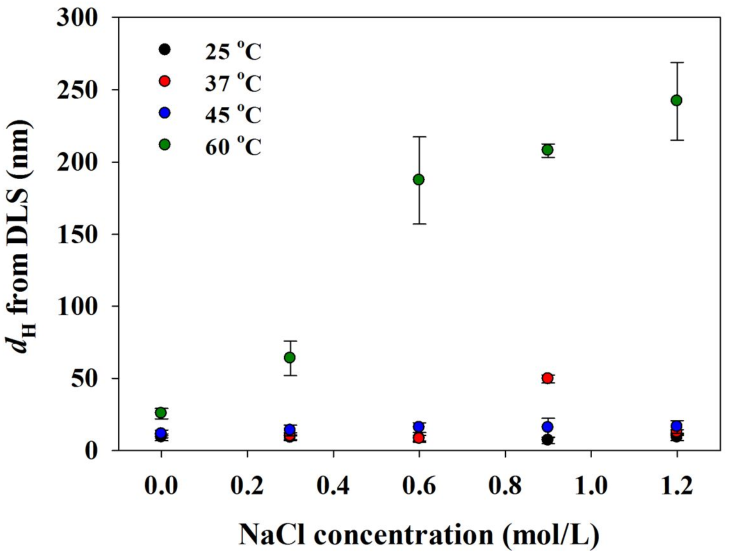

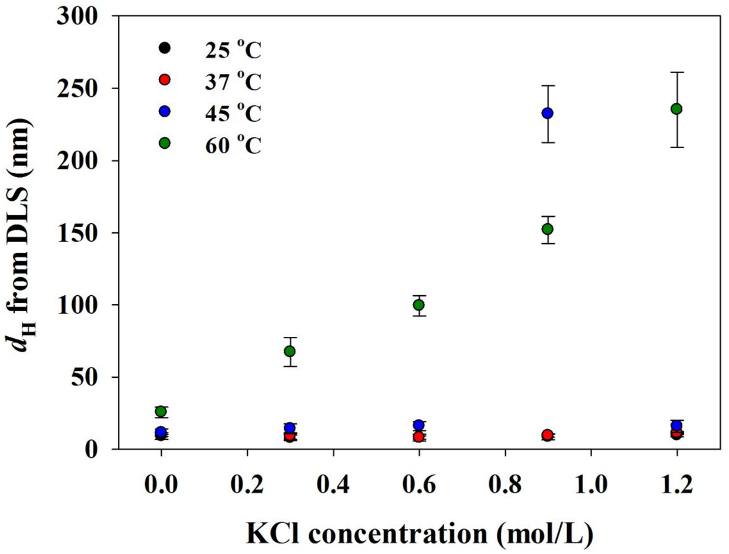

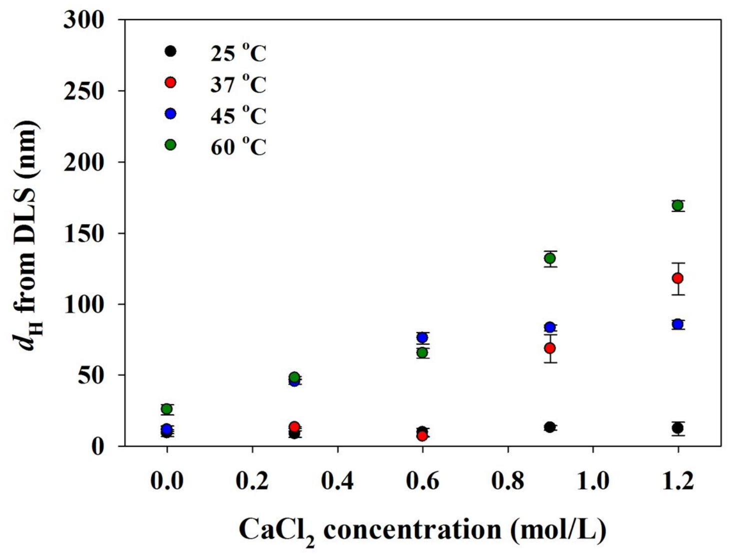
25 °C
37 °C
45 °C
60 °C
dH from DLS (nm)
CaCl2 concentration (mol/L)
0
50
100
150
200
250
300
0.0
0.2
0.4
0.6
0.8
1.0
1.2

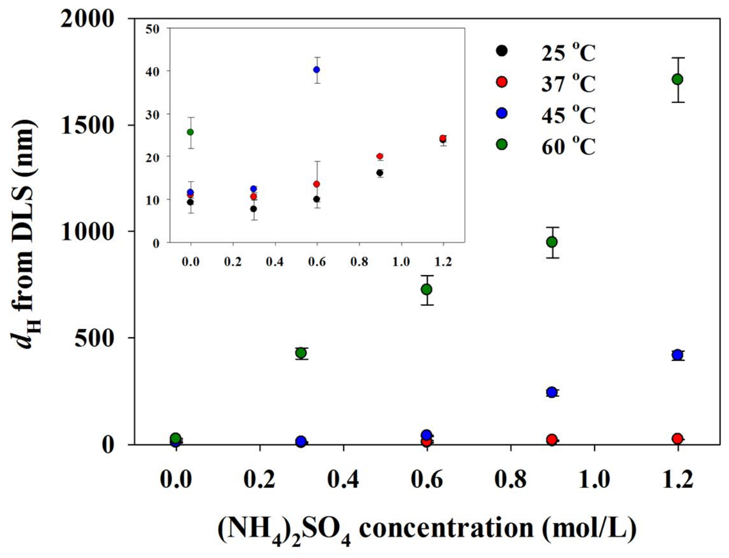
25 °C
37 °C
45 °C
60 °C
dH from DLS (nm)
(NH4)2SO4 concentration (mol/L)
0
500
1000
1500
2000
0.0
0.2
0.4
0.6
0.8
1.0
1.2

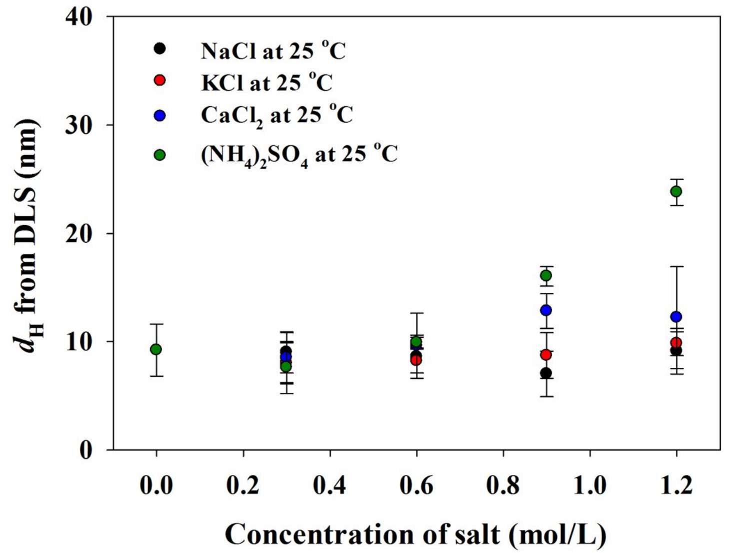
NaCl at 25 °C
KCl at 25 °C
CaCl2 at 25 °C
(NH4)2SO4 at 25 °C
dH from DLS (nm)
Concentration of salt (mol/L)
0
10
20
30
40
0.0
0.2
0.4
0.6
0.8
1.0
1.2

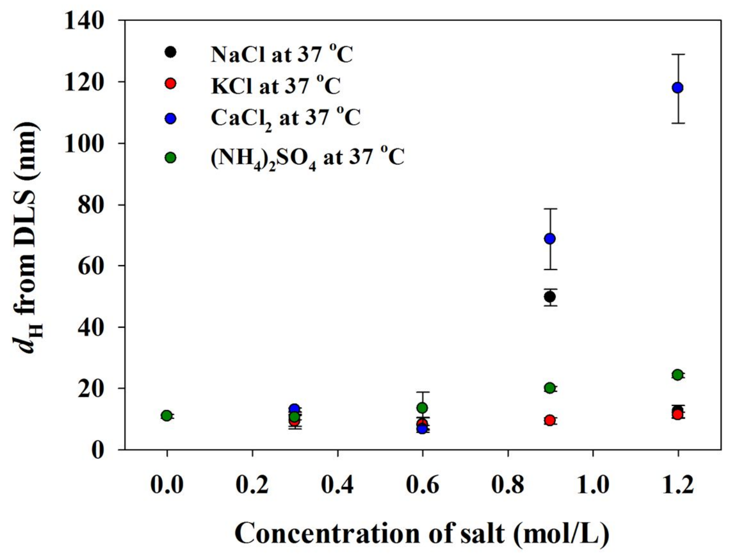

NaCl at 37 °C
KCl at 37 °C
$CaCl_2$ at 37 °C
$(NH_4)_2SO_4$ at 37 °C
d_H from DLS (nm)
Concentration of salt (mol/L)

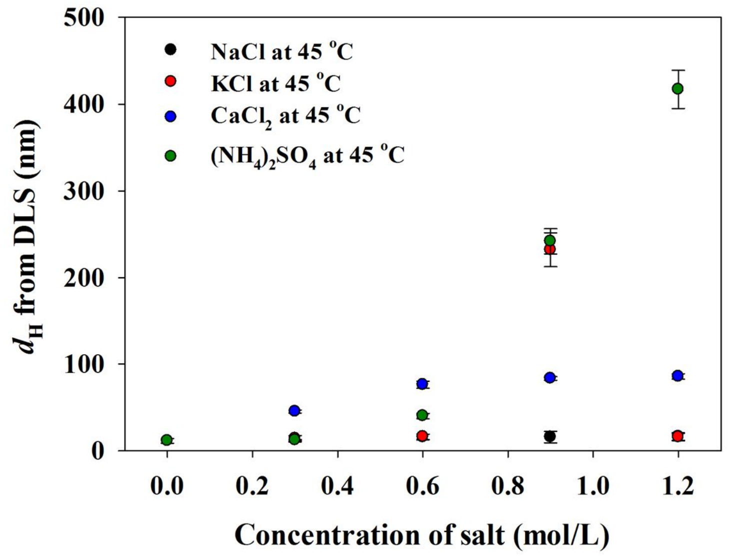

NaCl at 45 °C
KCl at 45 °C
$CaCl_2$ at 45 °C
$(NH_4)_2SO_4$ at 45 °C
d_H from DLS (nm)
Concentration of salt (mol/L)

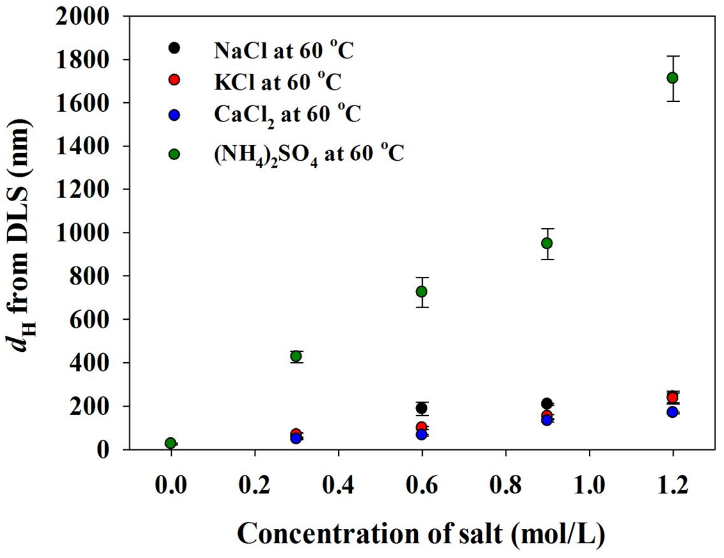

NaCl at 60 °C
KCl at 60 °C
$CaCl_2$ at 60 °C
$(NH_4)_2SO_4$ at 60 °C
d_H from DLS (nm)
Concentration of salt (mol/L)
